# Smart Web-Based Platform to Support Physical Rehabilitation

**DOI:** 10.3390/s18051344

**Published:** 2018-04-26

**Authors:** Yves Rybarczyk, Jan Kleine Deters, Clément Cointe, Danilo Esparza

**Affiliations:** 1 Intelligent & Interactive Lab (SI^2^ Lab), Universidad de Las Américas, Quito 170124, Ecuador; wilmer.esparza@udla.edu.ec; 2 Department of Electrical Engineering, CTS/UNINOVA, Nova University of Lisbon, 2829-516 Monte de Caparica, Portugal; 3 Faculty of Electrical Engineering, University of Twente, 217 7500 Enschede, The Netherlands; j.kleinedeters@utwente.nl; 4 Ecole Normale Supérieure de Paris-Saclay, 94235 Cachan, France; clement.cointe@ens-cachan.fr

**Keywords:** telemedicine, motor rehabilitation, motion assessment, natural user interface, Hidden Markov Model

## Abstract

The enhancement of ubiquitous and pervasive computing brings new perspectives in medical rehabilitation. In that sense, the present study proposes a smart, web-based platform to promote the reeducation of patients after hip replacement surgery. This project focuses on two fundamental aspects in the development of a suitable tele-rehabilitation application, which are: (i) being based on an affordable technology, and (ii) providing the patients with a real-time assessment of the correctness of their movements. A probabilistic approach based on the development and training of ten Hidden Markov Models (HMMs) is used to discriminate in real time the main faults in the execution of the therapeutic exercises. Two experiments are designed to evaluate the precision of the algorithm for classifying movements performed in the laboratory and clinical settings, respectively. A comparative analysis of the data shows that the models are as reliable as the physiotherapists to discriminate and identify the motion errors. The results are discussed in terms of the required setup for a successful application in the field and further implementations to improve the accuracy and usability of the system.

## 1. Introduction

A current trend in rehabilitation medicine is the concept of a remote therapy system [1,2]. This concept consists of enabling patients to carry out part of the rehabilitation at home and to communicate through the Internet the evolution of the recovery process. The implementation of such a technology is justified by medical (improvement of the recovery process by the possibility to perform rehabilitation exercises more frequently), economic (reduction of the number of medical appointments and the time patients spend at the hospital), mobility (diminution of the transportation to and from the hospital) and ethics (healthcare democratization and increased empowerment of the patient) purposes [3,4]. Nevertheless, the fact that the patients can perform the rehabilitation exercises by themselves and without the supervision of a therapist raises an issue regarding the correctness of the therapeutic movement [5,6].

The present study exposes a web-based platform for physical tele-rehabilitation for patients after hip arthroplasty surgery. This orthopedic procedure is an excellent case study, because it involves people that are limited in their mobility and who need a postoperative functional rehabilitation program to recover strength and joint mobility. The proposed approach considers two fundamental conditions for the development of a suitable tele-rehabilitation platform [7,8]. First, the system makes use of a low-cost motion capture device, in order to be economically viable. Second, the platform integrates an artificial intelligent module that automatically assesses the correctness of the executed movement to provide the patient with real-time feedback.

The remainder of the manuscript is organized into six parts. Section 2 presents related work in tele-rehabilitation systems. Section 3 is a general description of the web-based platform (frontend and backend). Section 4 focuses on the used method to develop the assessment module, which is based on Hidden Markov Models (HMMs). Section 5 consists of a laboratory experiment that tests the reliability of the HMMs to discriminate between correctly and incorrectly performed movements, and by comparison with the assessment made by therapists. Section 6 is a similar experiment carried out in clinical conditions and with patients after hip replacement surgery. Finally, the last section discusses (i) the results of the automatic assessment and possible improvements of the algorithm; (ii) the required setup for a reliable classification of the movements in real conditions; and (iii) a user-friendly application to enable the therapists to generate assessment models on any kind of new exercises.

## 2. Related Work

Table 1 presents the main related studies and approaches to build a physical tele-rehabilitation system. First of all, the motion capture can be based on inertial wearable sensors or visual sensors. Fortino and Gravina [9] propose a cloud-assisted wearable system (Rehab-aaService) that enables a general motor rehabilitation, even if the system is optimized for the upper limbs. The platform is scalable and can be integrated into a body sensor network (BodyCloud) [10] for the monitoring of different physiological parameters. However, the system does not have an artificial intelligent module that allows for a rigorous assessment of the rehabilitation exercises. In addition, the wearable devices require the inertial sensors to be precisely placed on the body and/or to involve a calibration stage. Thus, another approach consists of using a vision-based motion capture. This system also presents certain limitations, such as the occlusion problem, but it has the advantage to provide an easy setup. The occlusion can be overcome (at least partially) by asking the individuals to change the orientation of their body according to the plane in which the movement is performed. Most of the systems use the Kinect, because it is an affordable piece of equipment and its accuracy is good enough for functional assessment activities [11]. An avatar evolving in a serious game is usually used to motivate the user to regularly practice their therapeutic exercises [12]. However, it is very rare to find a system that provides both an attractive virtual environment and an algorithm to assess the quality of the rehabilitation movements executed by the patients. Instead of evaluating a spontaneous movement, other studies propose to guide the gesture through visual [13] or haptic [14] feedback, in order to avoid wrong motions. The disadvantage of these approaches is to induce a too stereotyped movement, which reduces the functional benefit of the rehabilitation. So, the technological implementation of an appropriate program of motor rehabilitation must involve an expert system that could substitute the therapist and provide the patient with feedback. Recent studies applied algorithms based on Dynamic Time Warping [15] and fuzzy logic [16] for the recognition of therapeutic movements and the diagnosis of physical impairments, respectively. Nevertheless, the current systems are only able to discriminate between a correct and an incorrect gesture, but they cannot give a targeted feedback on the type of error when a movement is wrongly executed. To get such a feedback, it seems necessary to build a model of the therapeutic exercises as suggested by [17], who propose a theoretical modeling in UML for the reeducation of the upper limbs. Our work proposes a more advanced approach, since we developed and implemented a statistical model to assess the rehabilitation movements, which can be applied on any part of the body (upper and lower limbs) and can precisely identify the cause of a bad performance to provide the users of the platform with comprehensive feedback regarding the corrections to be made to the gesture.

## 3. Web Platform

### 3.1. Global Architecture

**Figure 1:**
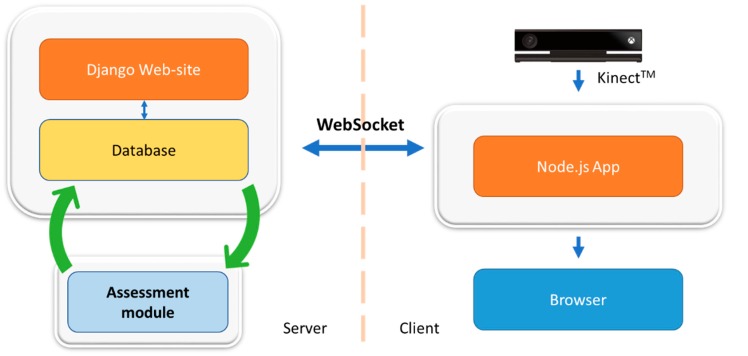
Platform architecture.

One of the main goals of the web application is to be easy to use for patients and physiotherapists. The rehabilitation platform is supported by a client-server architecture (Figure 1). The server contains the remote application and the associated database. The user’s interface enables patients and therapists to manage the display and the use of the Kinect data. The client-server connection is established via Web Socket as communication protocol.

#### 3.1.1. Django Website Server

The website is based on Django, a web framework written in Python. For an effective development, it was decided to use Django CMS, a content management system that simplifies the implementation of the web application.

#### 3.1.2. Client Side

**Figure 2:**
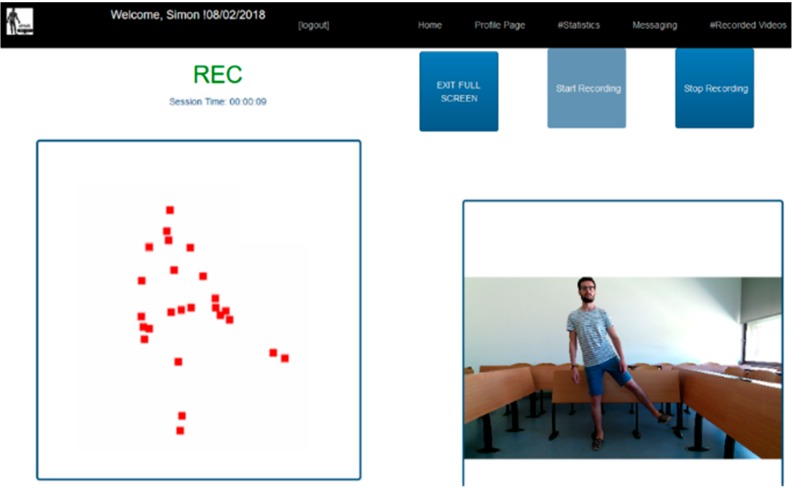
Patient’s interface during the practice of a rehabilitation exercise.

The Kinect camera is used to capture the movements performed by the patient. A network application is launched on the patient’s computer, in order to send the data from the Kinect to the Django website. To perform an exercise and to use the Kinect data, a gateway between the Kinect and the server is necessary to manage the data flow. This application launched in the backend of the system must be able to retrieve the data sent by the Kinect and create a link with the server to send them (Network communication). Node.js, a JavaScript run-time environment, is used for this part. It allows the creation of an application coded in JavaScript and HTML; and the communication with the Django server. The backend manages the data from the Kinect and generates an HTML page for the browser. Node.js was chosen for its wide range of modules, and more specifically the library Kinect2, which simplifies the communication with the Kinect. The library http is used to start the application. Figure 2 shows an example of the JavaScript application created to display the avatar of the patient in a browser. The avatar (point-light representation) appears in a window on the left side of the graphic user interface. A demonstration video of the exercise is displayed on the right side of the interface. Two buttons on the top-right corner allow the patient to start (after 5 s countdown) and stop the recording of the exercise, respectively.

#### 3.1.3. Web Socket

Node.js includes a library named Socket.IO and allows the deployment of a real-time bidirectional event-based communication. To do so, the destination address of the data (the address of the server hosting the website) must be declared in the Node.js application, and the IP address of the sender must be declared in the Django website. Through this method, the library Socket.IO for Python and the one for Node.js are able to communicate by using a function call.

### 3.2. Users Interface

The website can accommodate different types of users, such as patients and therapists. The functionalities are singular for these two kinds of users. The rehabilitation is divided into several parts, called stages. Each stage corresponds to a different level in terms of the intensity of: (i) Range of Movement (ROM); (ii) Stretching; (iii) Force; and (iv) Walking. The transition from one stage to another is allowed when the patient completes correctly all the exercises of the current stage and receives the physiotherapist’s consent. In other words, the physiotherapist only can unlock a stage according to the progress of the patient.

#### 3.2.1. Patient Interface

**Figure 3:**
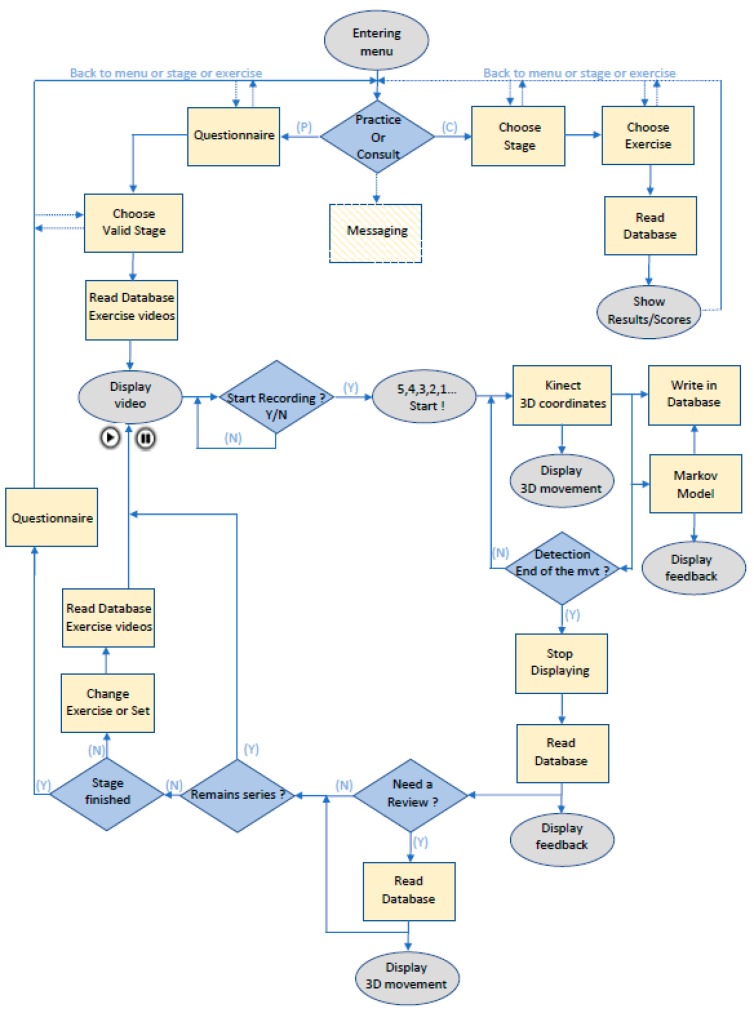
Workflow of the patient interface.

Figure 3 shows the workflow of the patient interface. The patients have the choice to practice exercises, consult their performances, or send a message to the health professionals.

**Practice.** Before the rehabilitation begins, patients must answer a questionnaire, which evaluates their ability to complete the exercises. They must self-assess pain levels, skin problems, and potential edemas. If the questionnaire outputs a low score, the patients are impeded to perform the physical exercises. In the opposite case, the patients can proceed with the rehabilitation protocol. They must choose an available stage and the various associated exercises. The reeducation program is divided into different items. First, they have the option to watch an explanation of the movements. After the exercise is starting, the user’s avatar and a real-time feedback on the quality of the movement is displayed on the screen (see, Figure 2). The 3D joint coordinates received from the Kinect and the assessment associated are saved into the database. At the end of the exercise, the patients can review their movements and have a detailed feedback on their performance. The same questionnaire as previously mentioned is asked to the user at the end of a practical session, in order to provide the therapist with information on the state of the patient after performing the physical exercises.

**Consult.** This section allows the patients to check the results and performances of the exercises they achieved.

**Messaging.** This section allows the patient to exchange messages with a health professional (questions, advice, etc.).

#### 3.2.2. Physiotherapist Interface

**Figure 4:**
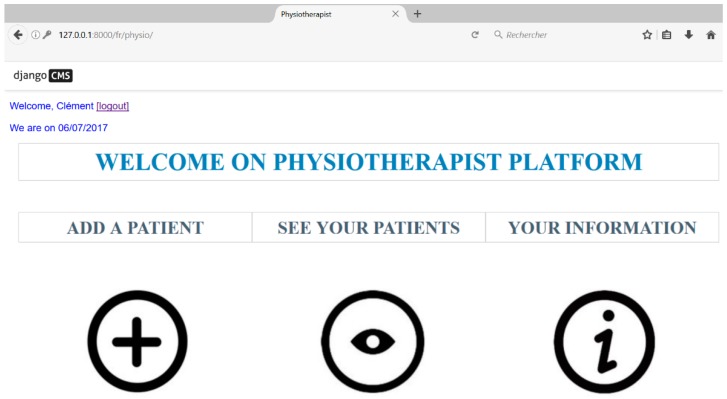
Physiotherapist interface (main menu).

Several functionalities are available for the health professionals. First, they have to create an account if they are not yet registered. After that, as shown in Figure 4, it is possible to: (i) add a new patient and/or update information on existing patients; (ii) monitor the performance, progress and movements of the patients; and (iii) send and/or receive messages from patients. Physiotherapists have also the possibility to update the intensity of the rehabilitation program.

### 3.3. Database Modelling

The database is directly associated with the Django website. Django CMS integrates a relational database management system named SQLite3, which is adequate for the requirements of the tele-rehabilitation platform.

#### 3.3.1. User Database

This database stores different information about the users, such as: name, e-mail, age, occupation, city, etc.

#### 3.3.2. Exercise Database

**Figure 5:**
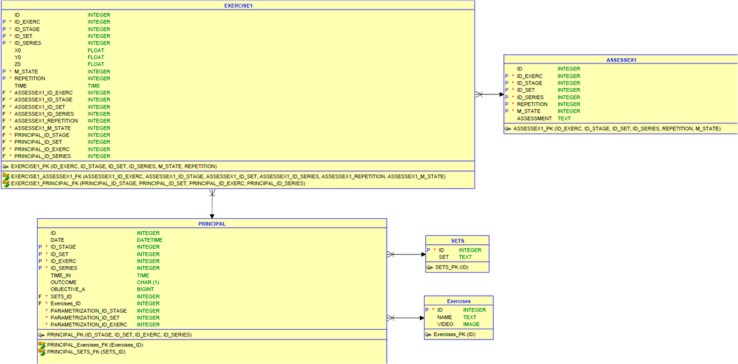
Architecture of the database.

The modelling of this database is as follows (see, Figure 5):

**Main table.** This table synthetizes the data about the exercises completed by the patient (date, kind of exercise, stage, completion time, etc.) and the corresponding outcome performance. During the execution of an exercise, fractions of the entire movement are assessed in real-time, in order to evaluate the correctness of the performed movement. Then, a single outcome (good, fair or bad) is produced based on the sum of these individual assessments (see, next section for more details on the assessment module).

**Exercises table.** This table, linked to the Main table through a 1:N relationship, provides details about each exercise and a demonstration video of the movement that must be performed.

Data exercise N* table. This table, linked to the Main table by a 1:N relationship, provides details about the movement performed during an exercise and stores the 3D joint coordinates captured by the Kinect. These data can be used to display a replay of the movement carried out by the patients, in order to provide the physiotherapist with an additional visualization (more ecologic) of the actual performance of the patient.

**Assessment table.** This table is linked to each Data Exercise N°* table, through a 1:N relationship, and stores the evaluation calculated by the assessment module for each part of the movement (see Figure 3, Markov Model box).

**Sets table.** This table, linked to the Main table by a 1:N relationship, describes the nature of the exercise (balance, coordination, ROM and force).

#### 3.3.3. Questionnaire Database

This database stores all the questionnaires answered by the patients and provides the physiotherapist with a medical history and a permanent monitoring of the patient’s condition.

## 4. Assessment Module

### 4.1. Hidden Markov Approach

To evaluate in real time the quality of the movement performed by the patient, an artificial intelligent program is developed to be integrated into the physical tele-rehabilitation platform as an assessment module. Our approach is based on the Hidden Markov Model, which is a probabilistic model that can be applied for time series analyses, such as gestures.

HMM can provide time-scale invariability when it comes to the recognition of temporal patterns. A HMM models real world data into so called ‘hidden states’. HMMs have been successfully applied in the domain of gesture recognition [18], speech and language processing [19], meteorological forecasting [20], stock market/economical trend analyses [21], among others. HMM is described in terms of probabilities. These are initial, transitional, and emission probabilities. Initial probabilities are the distribution of probabilities of ‘being in a state’ before a sequence is observed. Transitional probabilities are represented by a matrix, in which the probabilities indicate the possible changes from one state to another. Finally, the emission probabilities model the variance of each state’s associated values (mostly Gaussian Probability Density Functions or PDF) obtained from continuous variable observations. Estimating the model parameters is done by utilizing the Baum–Welch Expectation–Maximization (EM) algorithm, which is based on a forward–backward algorithm used in classifying Hidden Markov Chains [22,23]. The probabilities are calculated at any point of a sequence by inspecting previous observations, to find out how well the model describes the data, and following observations, to conclude how well the model predicts the rest of the sequence. This is an iterative process, in which the objective is to find an optimal solution (state sequence) for the HMM. This optimal sequence of states is inferred using the Viterbi algorithm. Also, the forward algorithm can be used to calculate the probability that a sequence is generated by a specifically trained HMM, making it applicable for classification. 

**Figure 6:**
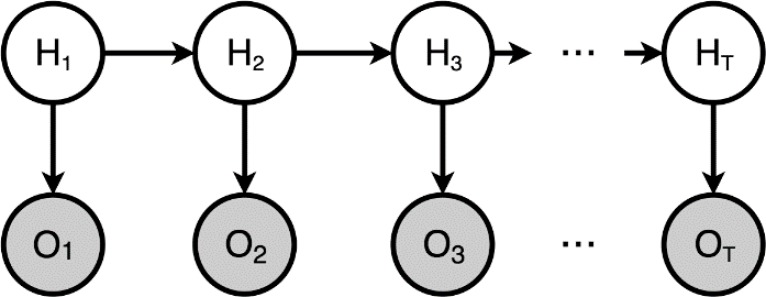
Trellis diagram of a Hidden Markov Approach (HMM).

HMMs are characterized by a set of real world observations (*o*_1_, *…*, *o*_n_) where (*o*_1_, *…*, *o*_n_) *ϵ X*(*Discrete*, *ℝ*, e.g.,). ‘States’ can be inferred from these observations. This set of ‘hidden states’ (*h*_1_, *…*, *h*_n_) takes values as follows: (*h*_1_, *…*, *h*_n_) *ϵ Y*(*ℤ*). The sequence of this set is represented in Figure 6. Since each state depends on its previous state and the observations are conditionally independent given the state, the joint probability *p*(*o*_1_, *…*, *o*_n_, *h*_1_, *…*, *h*_n_) is obtained by the factorization shown in (1).

(1) $$p\left(h_{1}\right)p\left(o_{1}|h_{1}\right)\overset{n}{\underset{i=2}{\prod }}p\left(h_{i}|h_{i-1}\right)p\left(o_{i}|h_{i}\right)\;$$

The transition probabilities include all the probabilities of changing from one hidden state (i) to another (*j*), such as *T_(j_*_,*k)*_ *= p(h_i_ = k | h_i−_*_1_ *= j) where j*, *k* *ϵ Y(ℤ*(1, *...*, n)) and result into a n × n sized matrix (transition matrix). The emission probabilities are described by *ε_l_(o) = p(o | h_i_ = l)* where *l* *ϵ Y*(*ℤ*(1, ..., n)) and correspond to the states. *o* *ϵ X*(*Discrete*, *ℝ*, e.g.,) and corresponds to the observed data. Each *ε_l_(o)* is a probability distribution function (PDF) of the observed data for *state l.* There are different distribution types depending on the values of *X*. A distribution type for these probabilities must be defined within the algorithm. For Discrete values, the distribution type could be a Probability Mass Function (PMF) or multinomial distribution, whereas for *o* *ϵ X(ℝ)* commonly used distribution types are Gaussian based (regular Gaussian emissions and Gaussian mixture emissions). Finally, the initial distribution is *π(l) = p(h_l_ = l)* where *l* *ϵ Y*(*z*(1, *...*, n)) (being a PMF) and is described in (2).

(2) $$\pi \left(h_{1}\right)\varepsilon_{h_{1}}\left(o_{1}\right)\overset{n}{\underset{i=2}{\prod }}T_{\left(h_{i-1},h_{i}\right)}\varepsilon_{h_{i}}\left(o_{i}\right)\;$$

Using the forward algorithm, it is possible to calculate the probability that a sequence is generated by a trained HMM. Calculating the probably for a sequence on N trained HMMs and choosing the highest probability provides us with a recognition and further assessment of the movement.

### 4.2. Feature Representation

**Figure 7:**
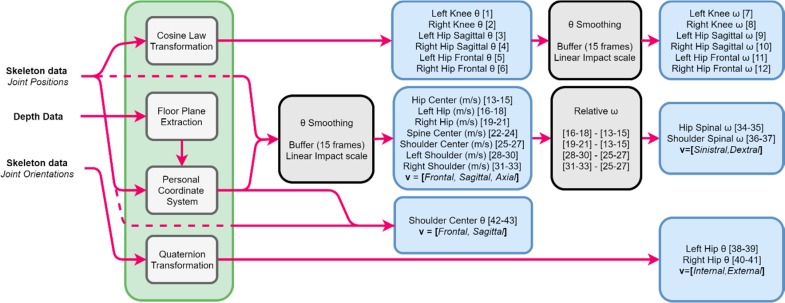
Proposed feature representation. The green box represents the required processing steps. The grey boxes indicate minor transformations. And the blue boxes are the sets of features. The arrows show the direction of the data flow.

The Kinect camera is used to extract the three-dimensional coordinates of the main body joints. Nevertheless, additional data are required for the feature representation. The joint positions only are not sufficient to create the motion representation. It is also necessary to consider the joint orientation data and the depth data. The joint orientation is a 4d vector (quaternion) containing the coefficients to calculate pitch, yaw and roll between two sets of coordinate systems (in this case the joint specific orientations). These rotations enable us to detect harmful movements. The depth data is needed to create the personal coordinate system so that motion can be expressed in terms of relative movement in the frontal, sagittal and horizontal planes. The proposed total feature representation is represented in Figure 7.

The transformation from joint positions to angles is one of the straight forward tasks and can be computed rapidly utilizing the cosine law (3). The absolute lengths between joints are calculated, in order to extract an angle as feature. The first features are created to represent the relative pose of the subject. These are features 1–6 as shown in Figure 7.

(3) $$Cos\;\left(A\right)=\;\frac{-a^{2}+b^{2}+c^{2}}{2bc}\;$$

**Figure 8:**
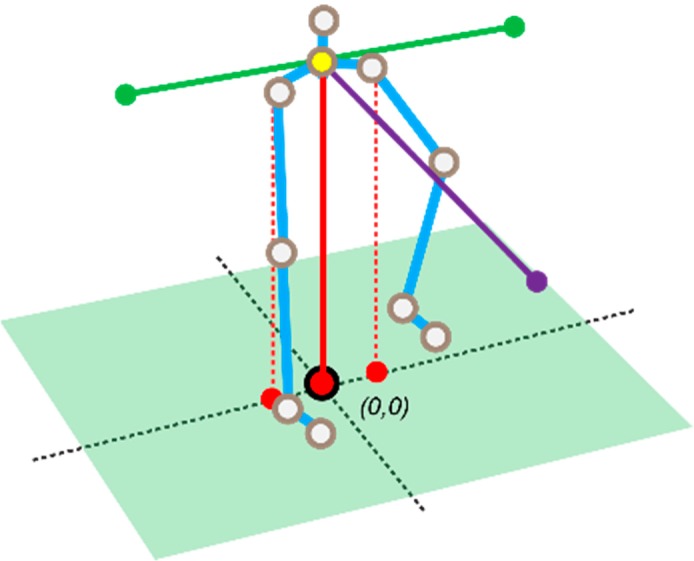
Vectors creating the personal coordinate system with the center floor point being the new origin. The cyan lines represent the lower limbs. The dotted lines indicate the additional vectors that are required to calculate hip movements in the sagittal plane.

Then, additional vectors are computed in a similar fashion, in order to represent hip rotations in the sagittal plane (Figure 8). With the floor plane orientation, the Personal Coordinate System (PCS) can be constructed. The additional data required in this step are the left and right hip joint position data. The vector that crosses both these points can be translated onto the reference point. Now at this point two vectors meet at the reference point and enable the third direction to be extracted by finding the perpendicular of these directions. This can be done by using the cross-product rule (4).

(4) $$A^{\to }\times \;B^{\to }=\left[a_{2}b_{3}-a_{3}b_{2},\;\;a_{3}b_{1}-a_{1}b_{3},\;\;a_{1}b_{2}-a_{2}b_{1}\right]\;$$

Using the newly found orientations, the absolute coordinates per joint can be translated into personal coordinates, where the origin is the floor crossing with the earlier mentioned scalar (Figure 8). Note that the PCS is rotation invariant so that movements can be expressed in terms of front/back, left/right and up/down. The speeds in the three directions are differences in orientation between consecutive frames. Here, the speed is calculated as an averaged sum of 15 frames (±0.25 s). The rotational values (features 34–37) are also calculated using the absolute values.

The rotations that need to be captured are those that resemble the external and internal rotation of the upper leg. Using the joint orientations, quaternion values can be transformed into different rotations (x, y, z of parent axis). These values are initially used in animations to easily recreate the rotation of limbs. A hierarchical bone structure and a coordinate system per joint enables the rotations to be extracted from the quaternion [24].

### 4.3. Trained HMMs

**Figure 9:**
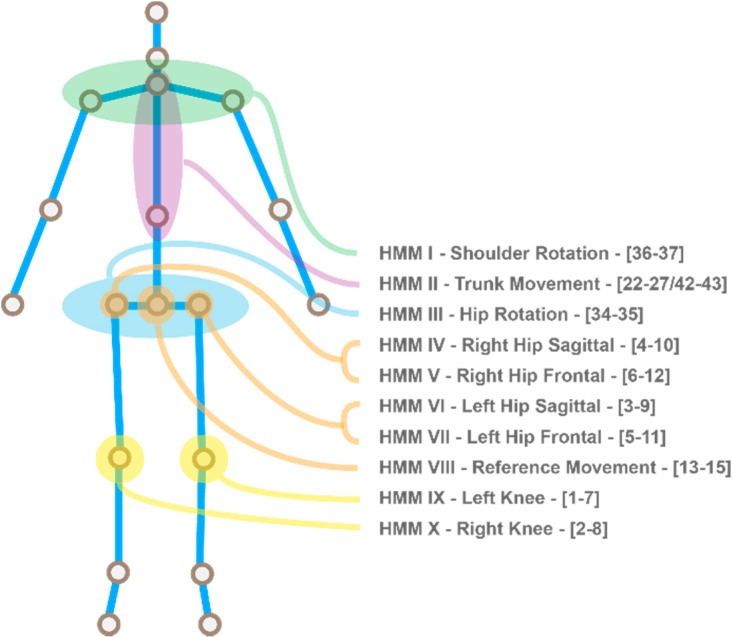
The 10 HMMs that are trained on specific features to identify a determined error in the movement (in brackets the used features per HMM).

The features are input into the learning parameters on the level that semantics comply with a therapist’s analysis of an exercise. Most coarse types of movements (with this semantic similarity) are incorporated into the HMM representation, where the restrictions of the Skeletonization algorithm are also taken into account (e.g., no trunk bending possible). This means that the earlier developed multi HMM assessment has advanced into a 10-fold semantic manifestation, which is represented in Figure 9. For each of these HMMs, two measures advance into the assessment blueprint. The forward probabilities are used with a sliding window to indicate the likelihood of each phenomenon within the movement. This pinpoints the location of an error, if this occurs in one of the HMM elements. Then, the state transition sequence is passed on to be analyzed on symmetrical values and coordination between the different HMM elements. When the forward pass provides a low likelihood, the corresponding state mean values (those containing directional speed) are compared in an absolute fashion to determine the direction of the error.

## 5. Experiment 1

An automatic assessment of the quality of the performed exercises is implemented according to a Hidden Markov Models (HMM) approach [25]. This technique provides insights into the ontological structure of the rehabilitation exercises and represents a probabilistic interpretation of the correctness of movement execution [22,26]. Here, the ontology of different compensatory movements associated with an exercise of hip abduction of the right leg are evaluated from an experiment on healthy participants. Compensation strategies are commonly used by patients to make exercises easier [27]. However, these compensatory movements limit functional improvements from therapy of the affected limb and can cause pain [28,29]. This is the reason why several motor rehabilitation support systems were interested in identifying and limiting these compensations, in order to recover a normal joint coordination for an appropriate use of the limb in everyday life activities [13,14]. The accuracy of the proposed algorithm is validated by a comparison with the assessment made simultaneously by physiotherapists, as it is suggested by other studies to evaluate e-rehabilitation systems [13,15].

### 5.1. Setup and Protocol

Nine subjects took part in the study. The experiment consisted of executing a rehabilitation exercise of hip abduction. This exercise was repeated eight times per participant. Simultaneously, five therapists rated the movement on three different aspects. These aspects were range of motion (ROM), coordination, and compensation. ROM corresponds to the amplitude of the movement. Coordination is related with the synchronicity of different body parts during the execution of an exercise. Compensations were defined as undesired movements, which were not expected to be performed. Subjects received feedback regarding the correctness of their performance after the fifth repetition. Then, each of the three-remaining repetitions was followed by systematic feedback from the therapists.

The representation of the Kinect’s skeletal data is based on the degrees of freedom per joints. The created feature vector contains four variables (Table 2). Two of these features are the absolute value of the linear speeds of the hip center joint and the shoulder center joint (features F0–F1, in Table 2). The speeds are calculated using a buffer of 15 frames (around 0.25 s) as a denoising method. The speed is the average of the values contained in the buffer. This buffer gets updated at every frame by discarding the oldest data point and adding the newest one. In this sense the data stream becomes available after the buffer is filled. Also, the angles of the upper legs in frontal planes (feature F3, in Table 2) are integrated into the model. The angles are calculated with the cosine law. At last, the speed paths of this last feature (feature F2, in Table 2) are created in the same fashion as stated previously.

### 5.2. Models Training

HMM is a probabilistic approach that aims to model a given signal into hidden states. These states represent an arbitrary decomposition of the whole movement into successive phases. For instance, the states for hip abduction of the right upper leg are: beginning pose (state 0), moving up (state 1), hold leg up (state 2), and leg down (state 3). Here, two HMMs are independently trained: (i) one on the movement of the trunk (HMM II, in Table 3); and (ii) another on the movement of the hip in the frontal plane (HMM V, in Table 3). Table 3 shows the feature set and the number of states used for training each HMM. The states are used in the next stage to create values to assess synchronicity and symmetry. For a determined individual, HMMs were trained by using the correct trials (according to the therapists labelling) of the other eight participants. Thus, a total of eight HMMs per subject were trained. Each model can predict the state of a specific joint or joint group (as the trunk in the skeleton representation is rigid). This is useful as a fault can occur in one of these groups only, while the rest can be correctly executed. In addition, every part of a movement can be correctly executed, but badly synchronized. Such structure enables us to identify if a movement in one joint initialized and stopped earlier or later, as state transitions are easy to obtain. The characteristic pattern of a movement expressed in states leads to the knowledge of which state is associated with the movement initialization and termination.

### 5.3. Results

Here, the performance of three subjects after the execution of a movement of hip abduction are presented. The result of the labelling shows a global consensus, but also some inconsistences between the therapists. Table 4 shows the labels for this sample of three participants. For each subject, the first row is the ROM rating, the second row is the coordination rating, and the third row is the compensation level.

The compensation is estimated through the symmetry of the movement, because it provides us with an insight on the motor control over the execution, which must be characterized by an even distribution of the agonist and antagonist muscular load in case of symmetry. It is first calculated by extracting the signal of the shoulder center joint on both terminations: beginning and end of the rest state (0).

**Figure 10:**
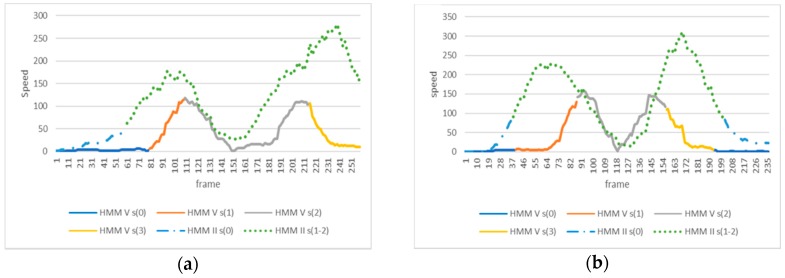
Speed path of the shoulder center (dotted lines) and angular speed of the right hip (continuous lines), for two different trials of an exercise of hip abduction: trial 7 of subject 2 (**a**) and trial 2 of subject 1 (**b**). The linear speed of the shoulder center is 1500 times magnified in this representation. The x-axis represents the number of frames, where every frame has a period of time of 1/60 s. The y-axis represents the velocity in m/s for the shoulder movement and θ/s for the hip movement.

As shown in Figure 10a,b, this processing provides a middle area (shown in green, HMM II) where the minimum value corresponds to the center of the total movement of the shoulder center. Then, the symmetry is expressed in differences in length (or number of frames) between the left and right side in relation to this minimum value. A ratio is obtained by dividing the time took to reach the maximum amplitude where speed ≈ 0 (inversion of the direction of the movement represented by the green valley in Figure 10a,b) and the time took to return to the initial pose (end of the movement). Table 5 shows these ratios. Values > 1 mean that returning to the initial pose took longer than reaching the maximum amplitude of the movement and inversely for ratios < 1.

The coordination is calculated by using the target movement (HMM V) and the relative shift in time of the movement of the trunk (HMM II). The shift in time is calculated by taking the midpoint of the sequenced state 2 (where maximum amplitude is reached) of HMM V and the relative difference in lengths of the shoulders clipping points on the left and the right side of this point. Figure 10a,b show an example of an incorrect and correct coordination of the movements, respectively. Figure 10a corresponds to the trial 7 of subject 2, which was classified by three therapists out of five as not perfectly coordinated (see Table 4). It can be noted on this trial a minor shift of the speed paths of the shoulder center to the left with respect to the angular speed of the hip abduction (ratio = 0.72, which is lower than the average value of 0.8 for this individual). In contrast, Figure 10b shows a trial that is almost perfectly synchronized (ratio = 0.94) as both valleys (movement of shoulder center and movement of abduction) are close to align.

## 6. Experiment 2

The second experiment is set up to study the applicability of the HMM models in assessing the quality of the self-rehabilitation movements in real-time and in clinical conditions. The quality is evaluated as correct or incorrect, with compensation faults that cover the most common errors during a regular rehabilitation program. Such an experiment is justified by the fact that unfortunately a large majority of the prototypes that aim to support physical rehabilitation are not tested on the end users [30].

### 6.1. Setup and Protocol

Two patients (74 and 77 years of age) and one healthy subject participated in this experiment. The patients were attending their regular session of physiotherapy for their rehabilitation. Both patients had undergone hip replacement surgery at their right hip. This surgery took place 1.5 and 3 weeks prior to the experiment (i.e., early stage recovery). All the participants were informed and gave their consent on the gathering of their exercise data and later use for scientific research purposes. Ethics clearance for this study was granted by the Nova University of Lisbon. Videos of the whole session were created as well as in depth images for further extraction of the skeleton and applying the input transformation into the desired feature representation. The participants were asked to perform five different exercises, which were repeated eight times (i.e., total of 120 recordings). The executed exercises were: hip abduction, hip extension, hip flexion in standing position, hip flexion in sitting position, and a three-step exercise (step forward followed by step to the side and later step backwards). Two therapists were asked to rate the quality of the movements performed by the subjects. Three aspects were evaluated: range of motion (ROM), compensation, and coordination. Table 3 describes the characteristics of the trained HMMs that were used in this experiment (HMM II and HMM V) to classify the movements of a patient and compare the results to the healthy subject. Then, the therapists’ evaluations were used to determine if the compensatory movements were correctly identified by the HMMs.

### 6.2. Results

#### 6.2.1. Conditioning

**Figure 11:**
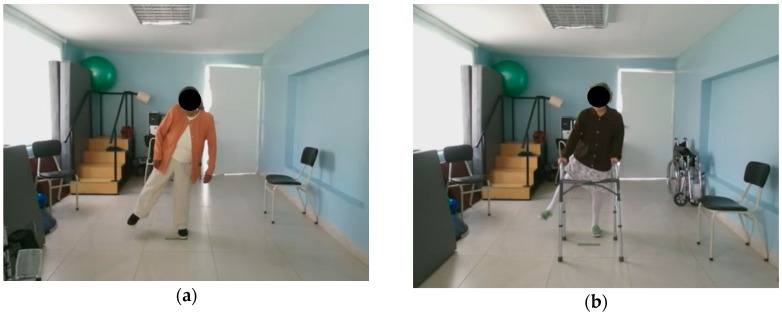
The two patients executing a therapeutic exercise of hip abduction: (**a**) is patient 1; (**b**) is patient 2.

As the patients were relatively old, the therapists demonstrated the exercises a couple of times with a clear and slow description of the action itself. The first patient had problems with memorizing the order of the three-step exercise, showing that cognitive abilities play an import role in the selection of suitable exercises in the rehabilitation plan. Both patients wore baggy clothing that did not afflict with the therapist’s assessment. However, it led the tracking of the skeleton to be slightly corrupted and created data that were unsuitable for the analysis of several movements executed by patient 1. The second patient needed to perform the exercises with a walking chair as she still had stability issues. This chair did not impact the skeleton extraction on the same level as the clothing did. This is the reason why the analyses will focus on patient 2. Figure 11 shows the patients performing an exercise. Minor feedback was provided after every execution. This feedback mostly consisted of reducing compensatory behaviors (e.g., stand up straight and let the force be applied from the hip).

#### 6.2.2. Classification Comparison

An analysis is performed on the executions of patient 2 regarding hip abduction. This is compared with the trials executed by the heathy subject. As shown in Table 6, both therapists agree on the fact that executions 4 and 5 have a lesser quality (i.e., containing compensation to some extend).

**Figure 12:**
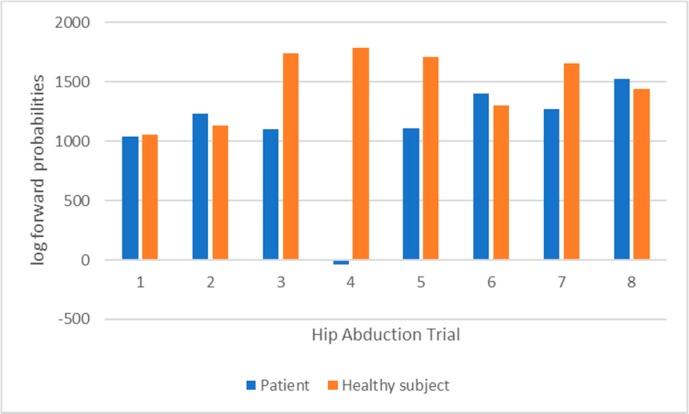
HMM II (trunk model) classification for the 8 trials of patient 2 (blue) and healthy subject (orange).

Figure 12 shows the classifications performed by the HMMs for both patient and healthy subject. The average probability of correct labelled exercises is 1261 for the patient vs. 1474 for the healthy subject, which indicates an average better execution by the healthy individual than the patient. The most relevant result of this analysis is the very low value (about 0) attributed by the HMM II to trial 4. It means that the model is able to identify that an error occurred in this trial. The fact that a similar assessment is made by the therapists (this trial received the lowest score) suggests that the automatic evaluation is as accurate as the human one.

#### 6.2.3. Error Analyses

**Figure 13:**
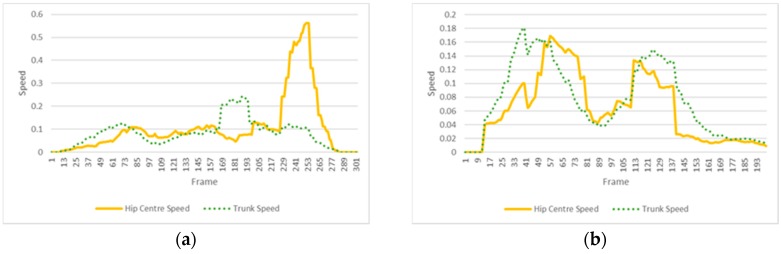
Trunk (green dotted lines) and hip (yellow unbroken lines) movements of patient 2 during a hip abduction exercise. (**a**) A wrong execution (trial 4), in which a compensation occurred at the end of the movement. (**b**) The example of a movement identified as correct (trial 3).

Further details on the compensation in executions 4 and 5 are provided in this section. The compensation is estimated through the symmetry of the movement, because it provides us with an insight on the motor control over the execution, which must be characterized by an even distribution of the agonist and antagonist muscular load in case of symmetry. This symmetry is defined by the difference in length (or number of frames) between the left and right side in relation to this minimum value. A ratio is obtained by dividing the time took to reach the maximum amplitude where speed ≈ 0 (inversion of the direction of the movement represented by the green valley in Figure 13, right panel) and the time took to return to the initial pose (end of the movement). Analyzing the video of trial 4 shows a clear compensation. This compensation was an uncontrolled deviation of the hip (center) moving to the left at the end of the movement. This is shown in Figure 13, where the yellow line represents the speed of the hip center during this trial (left panel), and for a correct execution (right panel) by the patient. This compensation can be referred to as adduction and occurred on the left hip. It suggests that the joint did not present the stability required to correctly perform the exercise, because the muscles of the opposite hip could not support the body while returning to the initial posture.

**Figure 14:**
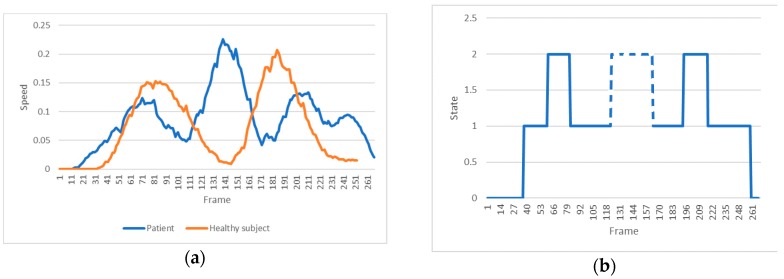
(**a**) Representation of the velocity of the trunk movement for a correct execution by the healthy subject (orange line) and an incorrect execution by the patient (blue line). (**b**) Description of the predicted state transitions for the trial 5 of patient 2, which is provided by the HMM II. To note the additional state transition (dashed line) caused by the compensatory behavior.

Trial 5 was classified as fair, only. Figure 14 (left panel) represents the velocity of the trunk movement for a correct (healthy subject) vs. an incorrect (trial 5 of patient 2) execution. An additional spike can be seen in the patient’s signal. The video shows that while approaching the maximal range within the execution the patient lost balance resulting in a minor fall. However, she was able to correct this in time and successfully terminated the exercise. This imbalance can be clearly seen when predicting the state transitions path (Figure 14, right panel). A correct execution should have two ‘bumps’, only. However, the HMM detects an extra ‘bump’ in the mid-section, which corresponds to an additional state transition into state 2 (broken line). This result demonstrates that the state transition predictions are good indicators of compensation.

## 7. Discussion

**Figure 15:**
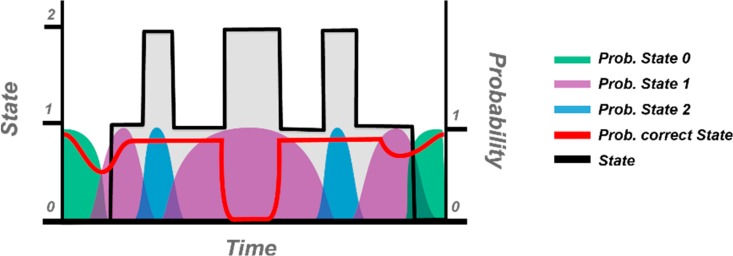
Versioned detection of being in an unlikely state (red line). The transition path of an incorrect execution (trial 5 by patient 2) is used as an example to illustrate the distribution over time of the expected current state. Here, an undesired state is clearly identified in the middle of the sequence.

This study demonstrates that HMMs permit a discrimination between accurately and wrongly executed movements of self-rehabilitation. The assessment module seems to capture to some extent the levels of synchronicity and compensation perceived by the therapists. In such an approach, the state transition sequences provide an important clue about the occurrence of compensations. Thus, it is imperative that the trained models learn the distribution of the states over time so that missing/added or elongated/shortened states are directly used as predictor of the correctness of the movement. Figure 15 shows an example of the probabilistic-based method applied in the HMM approach to detect a possible sequence mismatching. This technique of evaluation can also provide a qualitative insight regarding the stability and flexibility of the human body across the recovery process.

However, the algorithm could be substantially improved by including the total displacement of the trunk during the movement and a symmetric value for the distribution of the displacement, in which the total displacement would aid in detecting the allowed movements (not considered as compensation) and symmetry in displacement would provide an insight regarding the smoothness of the movements. In addition, the precision of the synchronicity could be increased by applying a curve fitting to the middle section of the speed paths (for shoulder and hip) and calculating the shift between the minimum of each curve. Also, more information on the compensatory movements could be obtained by determining the movement of the subjects regarding both the sagittal and the frontal planes. The evaluation of the force recovery was not developed in this study, since it is difficult to assess it using the Kinect. A possible option to overcome this limitation would be to implement a multimodal system using both a Kinect and a haptic device that could simulate the pressure the therapist applies to the limbs during a regular session [14].

From a computational point of view, the algorithm could be adapted to Hidden Semi-Markov Models (HSMMs), in which the sequences are modelled in terms of duration distributions [31]. This approach allows for a higher flexibility of the transition probability than in the HMMs, which should increase the classification accuracy [32]. It is also to mention the fact that the HMM predictions can sporadically contain noise. This issue was partially caused by the patient’s clothing that reduced the accurate detection of the skeleton. Thus, the use of tight clothes must be preferred and advised while patients perform exercises. The mentioned learned state duration distributions could be applied as a signal pre-processing to identify possible misclassifications caused by a noisy input, or to discard the exercise from evaluation.

It is important to highlight the fact that our approach is an extensive blueprint, which can be applied to a large scope of physical rehabilitations (including upper limbs). As the assessment is based on joint specific HMMs, the method can be crafted in numerous ways for implementation in any possible clinical setting or even field of motion analysis (e.g., sport, dancing, performance …). To do so, the only requirement is to collect new measures regarding the kinematic of the joint-based features involved in the specific exercises of each therapeutic program. Then, these data will be used to train pertinent HMMs that will enable the system to identify possible faults in the execution of the movement, such as reduced ROM, compensation strategies, problems of coordination … or any other incompliance that will disturb the recovery process. These errors could be handled by a pipeline of assessment, in which the most serious errors will be tackled primarily in the form of a targeted feedback for the patient.

**Figure 16:**
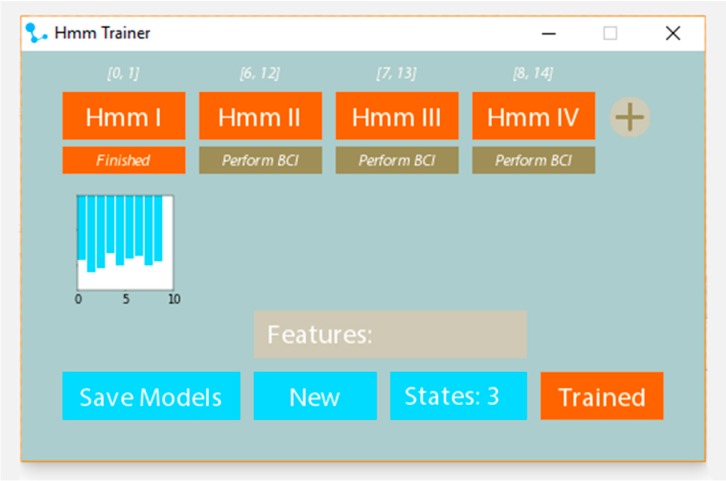
User graphic interface of the HMM trainer.

In order to facilitate this process, current work is focused on the development of an application to enable the therapists to easily create new assessment models that could be applied on any kind of rehabilitation exercises (see prototype interface in Figure 16). CSV files can be loaded by clicking an HMM button. Before doing so, the features that are going to be used in training need to be assigned by typing them in the features section. To analyze the optimal amount of states that a dataset requires, Bayesian Information Criteria (BIC) can be performed. This creates a score using cross validation for states 2–10 (as a HMM exists at least out of 2 states). The amount of states to be used during the training can be declared in the states section. When all the parameters are set, the training button appears and the training can take place. When this step is done, the models can be saved.

## Figures and Tables

**Table 1:** Characteristics of studies on tele-rehabilitation systems.

Studies	Type	Motion Capture	Movement Assessment	Feedback	Biological Features	Application	System Evaluation	Contribution/Results
Fortino and Gravina, 2015 [9]	Theoretical and applied research	Wearable device—Inertial sensors	Not presented	Visual—Avatar limb	Optimized for upper limbs	General motor rehabilitation	Not presented	Scalable system
Pedraza-Hueso et al., 2015 [12]	Applied research	Vision-based—Kinect	No	Visual—Avatar of a serious game	Movements of the whole body	Workout for elderly	Not presented	Attractive virtual environment
Da Gama et al., 2012 [13]	Applied research	Vision-based—Kinect	2D range of motion	Textual	Upper limbs	General motor rehabilitation	Physiotherapists and elderly	Avoiding wrong movements by guidance
Brokaw et al., 2013 [14]	Experimental research	Kinect and robotic system	By comparison with a reference	Haptic	Upper limbs	Stroke rehabilitation	One health subject	Multimodal interaction
Antón et al., 2015 [15]	Applied research	Vision-based—Kinect	Dynamic Time Warping	No	Posture and movements	General motor rehabilitation	Compared with therapists’ assessment	Accurate discrimination of the movements
Gal et al., 2015 [16]	Applied research	Vision based—Kinect	Dynamic Time Warping and Fuzzy Logic	Textual	Posture and motion ranges	Diagnostic of physical impairments	Not on real patients	Tested on healthy subjects
López-Jaquero et al., 2016 [17]	Theoretical research	Natural User Interface—Kinect	Not presented	Not presented	Body joints	Upper limbs rehabilitation	Not presented	Modelling of the therapeutic movements

**Table 2:** Feature vector.

F0	F1	F2	F3
Hip center speed (m/s)	Shoulder center speed (m/s)	Right hip speed (θ/s) Frontal plane	Right hip angle (θ) Frontal plane

**Table 3:** Feature sets and amount of states.

	HMM II	HMM V
**Features**	- Shoulder center (m/s) - Hip center (m/s)	- Right hip frontal (θ/s) - Right hip frontal (θ)
**Amount of States**	3	4
**Name**	Trunk Movement	Right Hip Frontal

**Table 4:** Labels (G for ‘good’, F for ‘fair’, and B for ‘bad’) for a sample of three subjects. The digits correspond to the Id number of the trial.

Subjects	Movement	Therapist 1			Therapist 2			Therapist 3			Therapist 4			Therapist 5		
	Parameters	G	F	B	G	F	B	G	F	B	G	F	B	G	F	B
**1**	ROM	1–8			1–8			1–8			1–8			1–8		
	Coordin.	1–8			1–8			1–8			1–8			1–8		
	Compens.	1–8			1–8			1–8			1–8			1–8		
**2**	ROM	1–8			1–8			1–8			1–8			1–8		
	Coordin.	1–6 8	7		1, 5, 8	2–4 6, 7		1–5 7, 8	6		3, 4 6, 8	1, 2 5, 7		1–8		
	Compens.	1, 3–5 7, 8	2, 6		1–8	4		2–6 8	1, 7		3–5 7, 8	1,2 6		2–4 6, 8	1, 5 7	
**3**	ROM	1–8			1–8			1–8			2–8	1		1–8		
	Coordin.	1–8			1, 3–5 7, 8	2, 6		1–8			2, 4 6, 8	1, 3 5, 7		1, 2 4–8	3	
	Compens.	1, 3–8	2		1, 3–8	2		2 6–8	1 3–5		2, 4 6, 8	1, 3 5, 7		2–4 7, 8	1, 5 6	

**Table 5:** Synchronicity between shoulder movement and symmetry of shoulder movement. Values close to 1 resemble a perfect synchronicity or symmetry. For synchronicity, values < 1 mean that the shoulders moved before the hip abduction and values > 1 mean that the shoulder center moved after the hip abduction. For symmetry, scores < 1 mean the shoulder center reached the maximum amplitude faster than it returned to the beginning pose and the opposite for scores > 1. For values > 1 an inverse value (1/value) is given in parenthesis, in order to calculate the average value for synchronicity and symmetry.

**Synchronicity**	**Subject 1**	**Subject 2**	**Subject 3**
**Trial 1**	0.93	0.79	0.95
**Trial 2**	1.06 (0.94)	1.16 (0.86)	0.74
**Trial 3**	0.86	0.91	1.21 (0.82)
**Trial 4**	0.73	0.61	0.64
**Trial 5**	1.54 (0.65)	0.74	0.73
**Trial 6**	0.93	0.93	0.71
**Trial 7**	0.74	0.72	2.14 (0.46)
**Trial 8**	1.01 (0.99)	0.84	0.87
**AVERAGE**	**0.85**	**0.8**	**0.74**
**Symmetry**	**Subject 1**	**Subject 2**	**Subject 3**
**Trial 1**	1.09 (0.92)	0.79	0.95
**Trial 2**	1.29 (0.78)	1	0.94
**Trial 3**	0.82	0.93	1.4 (0.71)
**Trial 4**	0.85	0.6	0.87
**Trial 5**	1.54 (0.65)	0.78	0.82
**Trial 6**	1.01 (0.99)	0.83	0.76
**Trial 7**	0.78	0.68	3.4 (0.29)
**Trial 8**	1.16 (0.86)	0.76	1
**AVERAGE**	**0.83**	**0.79**	**0.79**

**Table 6:** Therapist labelling of hip abduction for each trial (1–8).

	Therapist 1			Therapist 2		
	Good	Fair	Bad	Good	Fair	Bad
ROM		1–8		7, 8	1–6	
Coordination	1–6, 8	7		1–8		
Compensation	1–3, 6–8	5	4	1–3, 6–8	5	4
